# The Relationship Between Tumor Budding, Tumor Microenvironment, and Survival in Patients with Primary Operable Colorectal Cancer

**DOI:** 10.1245/s10434-019-07931-6

**Published:** 2019-10-11

**Authors:** Hester C. van Wyk, Antonia Roseweir, Peter Alexander, James H. Park, Paul G. Horgan, Donald C. McMillan, Joanne Edwards

**Affiliations:** 1 Academic Unit of Surgery, College of Medical, Veterinary and Life of Sciences, University of Glasgow, Glasgow, UK; 2 Unit of Gastrointestinal Cancer and Molecular Pathology, Institute of Cancer Sciences, College of Medical, Veterinary and Life Sciences, University of Glasgow, Glasgow, UK

## Abstract

**Background:**

Tumor budding is an independent prognostic factor in colorectal cancer (CRC) and has recently been well-defined by the International Tumour Budding Consensus Conference (ITBCC).

**Objective:**

The aim of the present study was to use the ITBCC budding evaluation method to examine the relationship between tumor budding, tumor factors, tumor microenvironment, and survival in patients with primary operable CRC.

**Methods:**

Hematoxylin and eosin-stained slides of 952 CRC patients diagnosed between 1997 and 2007 were evaluated for tumor budding according to the ITBCC criteria. The tumor microenvironment was evaluated using tumor stroma percentage (TSP) and Klintrup–Makinen (KM) grade to assess the tumor inflammatory cell infiltrate.

**Results:**

High budding (*n* = 268, 28%) was significantly associated with TNM stage (*p* < 0.001), competent mismatch repair (MMR; *p* < 0.05), venous invasion (*p* < 0.001), weak KM grade (*p* < 0.001), high TSP (*p* < 0.001), and reduced cancer-specific survival (CSS) (hazard ratio 8.68, 95% confidence interval 6.30–11.97; *p* < 0.001). Tumor budding effectively stratifies CSS stage T1 through to T4 (all *p* < 0.05) independent of associated factors.

**Conclusions:**

Tumor budding effectively stratifies patients’ survival in primary operable CRC independent of other phenotypic features. In particular, the combination of T stage and budding should form the basis of a new staging system for primary operable CRC.

Tumor budding has been defined as a single tumor cell or small cluster of four or fewer tumor cells at the invasive front^12^,^18^ and should be considered a promising and strong prognostic factor in colorectal cancer (CRC).^19^ Widespread reporting of tumor budding has not progressed to routine clinical practice due to a lack of consensus on scoring methods. However, routine reporting is now advocated by using the approach outlined by the International Tumour Budding Consensus Conference (ITBCC), with recommendations for the assessment and reporting of tumor budding in CRC.^6^

The ITBCC recommends that tumor budding should be included in future CRC guidelines and protocols and should be considered, along with other clinicopathological factors, in a multidisciplinary setting. The recent dataset for histopathological reporting of CRC by the royal pathologist stated that they would reconsider reporting tumor budding when new data become available.^4^

The tumor microenvironment also plays an important role in CRC outcomes. Marked peritumoral inflammation has been associated with favorable outcome,^3^,^14^ while the presence of a high tumor stroma percentage (TSP) has been validated as a stage-independent marker of reduced survival in patients with primary operable CRC.^7^,^8^. Both contribute to the development of a tumor microenvironment score that can potentially supplement the current TNM staging system.^9^

The aim of this study was to assess the proposed method by ITBCC in clinical practice and investigate the relationship between tumor budding and tumor factors, tumor microenvironment, and survival in primary operable CRC.

## Patients and Methods

### Patients

Patients were identified from a prospectively collected database of patients undergoing surgery for CRC between 1997 and 2007 at the Royal Infirmary, Western Infirmary and Stobhill Hospital, Glasgow. Any patient with a synchronous cancer, inflammatory bowel disease, and mortality within 30 days of surgery, who had received neoadjuvant therapy, was excluded. Furthermore, patients who had their disease managed entirely endoscopically, without formal colonic or rectal resection, were also excluded from the study.

Patients were staged according to the TNM criteria that was applicable at the time of surgery and to current practice in the UK.^4^ The West of Scotland Research Ethics Committee granted study approval. Patients were followed up for at least 5 years, and the date and cause of death were cross-checked with electronic case records. Cancer-specific survival (CSS) was measured from the date of surgery until the date of death from CRC.

### Routine Stains: Hematoxylin and Eosin and Elastica

For evaluation, two consecutive cut samples from each specimen were stained; one was routinely stained with hematoxylin and eosin (H&E), and the other was stained with Miller’s elastic stain (BDH, Poole, Dorset, UK) and counterstained with H&E. Routine pathological elastica staining was used to assess the presence of venous invasion.^13^. Only venous invasion was evaluated and no special staining was utilized to improve the assessment of lymphatic or perineural invasion.

### Assessment of Tumor Budding

**Fig. 1:**
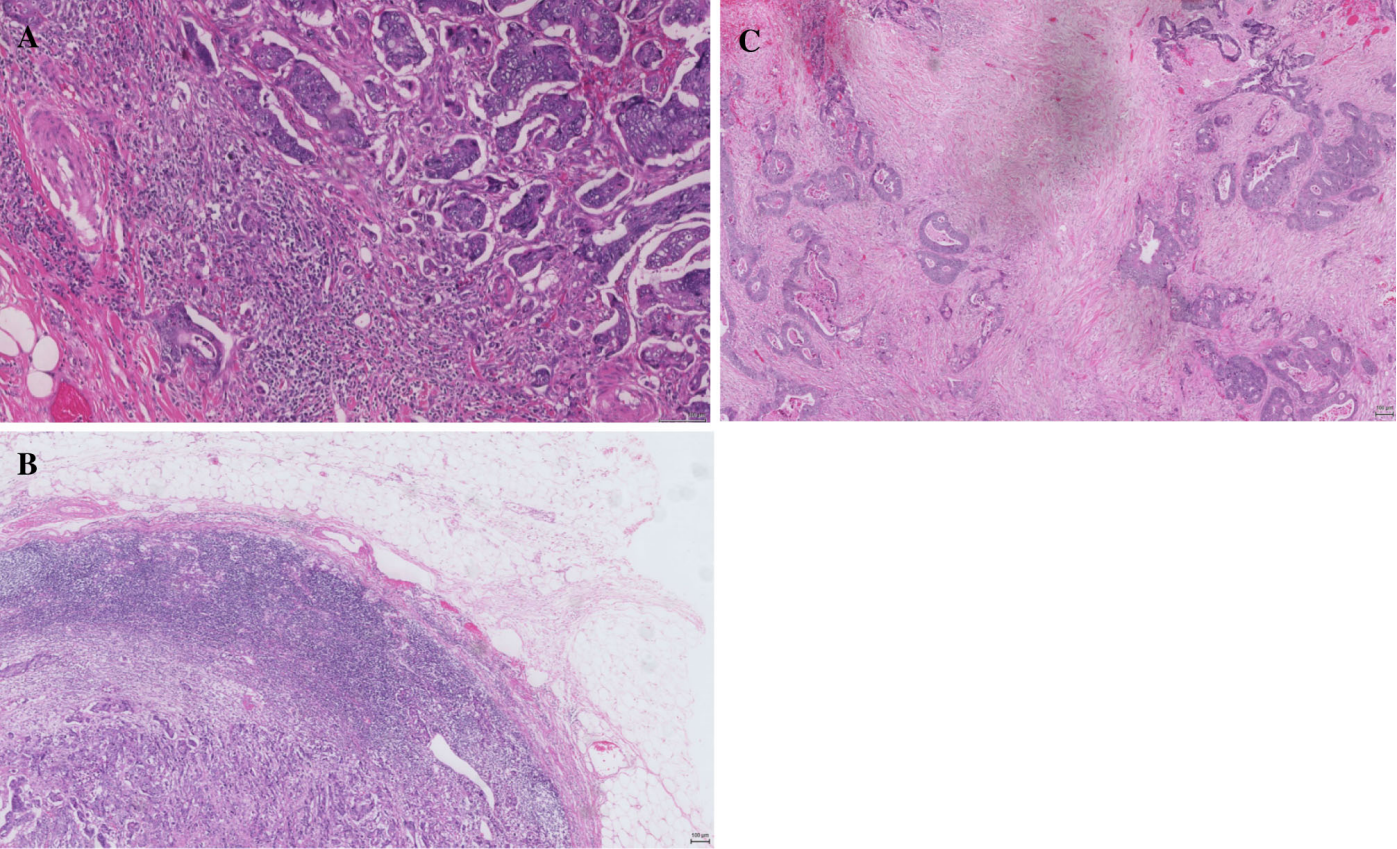
Example of **a** high tumor budding, **b** high inflammatory infiltrate (Klintrup–Makinen grade 4), and **c** high tumor stroma percentage

Tumor budding has been assessed on scanned H&E-stained slides in a single hotspot as defined by ITBCC. Tumor budding was counted on H&E slides, and was assessed in one hotspot (in a field measuring 0.785 mm^2^) at the invasive front. A two-tier system was used along with the budding count to facilitate risk stratification in CRC. Cut-offs used were: low, 0–9 buds; and high, ≥ 10 buds (Fig. 1a).

Tumor budding was evaluated by two different observers to ensure reliability, and co-scoring of randomly selected cases was carried out by HvW and PA. The interobserver intraclass correlation coefficient (ICCC) for the scores was 0.794 (*p* < 0.001).

### Evaluation of Tumor Inflammatory Cell Infiltrate and Tumor Stroma Percentage

*Klintrup–Makinen grade* was used to assess the generalized inflammatory infiltrate semi-quantitatively. Full H&E sections of the deepest point of tumor invasion were used, whereby inflammatory cell density at the invasive margin was graded using a 4-point scale; it was classified as low-grade (no increase or mild/patchy increase in inflammatory cells) or high-grade (prominent inflammatory reaction forming a band at the invasive margin, or florid cup-like infiltrate at the invasive edge with destruction of cancer cell islands), as previously described^3^,^14^ (Fig. 1b).

*Tumor stroma percentage* was assessed semi-quantitatively using full sections of the deepest point of tumor invasion; the proportion of stroma was calculated as a percentage of the visible field, excluding areas of mucin deposition or necrosis. Tumors were subsequently graded as low (50%) or high (> 50%) TSP as previously described^8^ (Fig. 1c).

### Assessment of Ki-67, Mismatch Repair (MMR) and BRAF Status

The Ki-67 proliferation index was assessed cohort using a threshold of 50%. A subset of patients in the full cohort underwent evaluation of the mismatch repair (MMR) and BRAF statuses. Using immunohistochemistry, a previously constructed tissue microarray comprising cores of formalin-fixed paraffin-embedded cancer tissue was used to assess MMR and BRAF status. Immunohistochemistry for MMR status has been previously described.^10^ MMR protein expression was reported as MMR-competent or -deficient, by a single blinded observer. The assessment of BRAF status was performed as previously described by our group^11^ and therefore the results were incorporated in our study. BRAF V600E mutation was reported as absent or present by a single blinded observer.

### Statistical Analysis

Interrelationships between tumor location, clinicopathological characteristics, and measures of the tumor microenvironment were analyzed using the Chi square test. Five-year CSS and overall survial was examined using Kaplan–Meier log-rank survival analysis and univariate Cox proportional hazards regression to calculate hazard ratios and 95% confidence intervals. Variables found to be statistically significant (*p* < 0.05) on univariate analysis were entered into a Cox regression multivariate model using a backward conditional method. A *p* value < 0.05 was significant. Analyses were performed using SPSS software version 24 (IBM SPSS, Armonk, NY, USA).

## Results

**Fig. 2:**
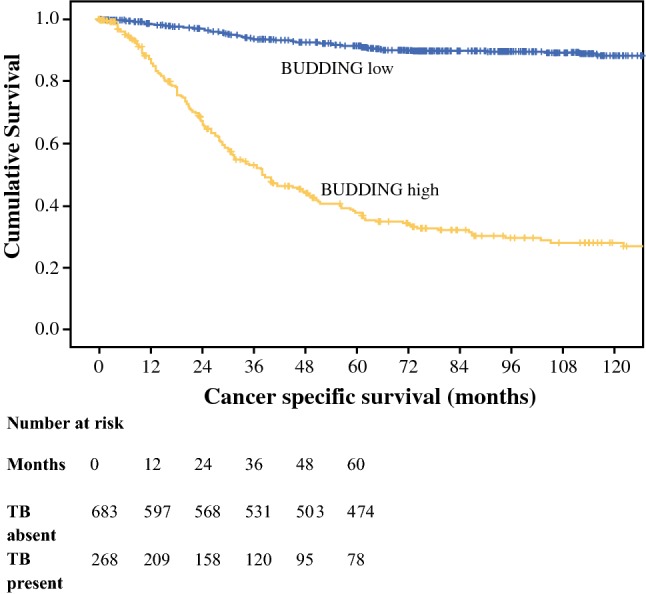
Relationship between low- and high-grade tumor budding and cancer-specific survival in patients with primary operable colorectal cancer (*p* < 0.001). *TB* tumor budding

The cohort consisted of 952 patients. Clinical and pathological features are shown in Table 1. Two-thirds of patients included were over the age of 65 years, and 52% were male. Overall, 131 (14%) patients had TNM stage I disease, 445 (47%) had stage II disease, 355 (37%) had stage III disease, and 21 (2%) had stage IV disease; 713 (75%) patients had colon tumors and 239 (25%) had rectal cancer. Venous invasion was present in 321 (34%) tumors, and tumor necrosis was present in 362 (38%) tumors. High-grade tumor budding was present in 28% of tumors, a low-grade inflammatory cell infiltrate was present in 69% of tumors, and 25% of tumors had a high TSP. MMR deficiency was identified in 17% of patients, and 20% of patients had BRAF V600E mutations. The median follow-up for patients was 11.7 years (range 6.4–16.3), with 226 cancer deaths and 271 non-cancer deaths. On univariate survival analysis, T stage (*p* < 0.001), N stage (*p* < 0.001), TNM stage (*p* < 0.001), Ki-67 proliferation index (*p* < 0.001), MMR (*p* ≤ 0.001), venous invasion (*p* < 0.001), Klintrup–Makinen (KM) grade (*p* < 0.001), TSP (*p* < 0.001), and tumor budding (*p* < 0.001) (Fig. 2) were significantly associated with CSS.

**Table 1:** Clinicopathological features of patients with primary operable colorectal cancer and cancer-specific survival

	*N* (%) [952 patients]	Univariate analysis [CSS]	*p* value
*Host characteristics*			
Age (years)		1.00 (0.76–1.32)	0.984
< 65	297 (31)		
> 65	655 (69)		
Sex		1.20 (0.92–1.56)	0.176
Female	456 (48)		
Male	496 (52)		
Adjuvant therapy		1.03 (0.68–1.28)	0.859
Yes	105 (34)		
No	204 (64)		
*Tumor characteristics*			
Tumor site		1.18 (0.89–1.58)	0.247
Colon	713 (75)		
Rectum	239 (25)		
Tstage		1.97 (1.61–2.41)	< 0.001
1	40 (4)		
2	117 (12)		
3	514 (54)		
4	281 (30)		
N stage		2.09 (1.78–2.47)	< 0.001
0	582 (61)		
1	253 (27)		
2	113 (12)		
TNM stage		2.95 (2.38–3.66)	< 0.001
1	131 (14)		
2	445 (47)		
3	355 (37)		
4	21 (2)		
Ki-67 proliferation index		0.60 (0.46–0.79)	< 0.001
Low	450 (48)		
High	491 (52)		
Mismatch repair status		0.64 (0.50–0.82)	0.001
Competent	763 (83)		
Deficient	162 (17)		
BRAF status		0.91 (0.65–1.28)	0.589
Low	735 (80)		
High	187 (20)		
Tumor necrosis		1.08 (0.82–1.41)	0.579
Low	581 (62)		
High	362 (38)		
Venous invasion		2.62 (2.02–3.41)	< 0.001
Absent	631 (66)		
Present	321 (34)		
Klintrup–Makinen grade		0.33 (0.23–0.46)	<0.001
Weak	651 (69)		
Strong	296 (31)		
Tumor stroma percentage		2.07 (1.58–2.74)	< 0.001
Low	704 (75)		
High	232 (25)		
Tumor budding		10.41 (7.76–13.96)	< 0.001
Low	684 (72)		
High	268 (28)		

*CSS* cancer-specific survival

The relationship between tumor budding, clinicopathological features of primary operable CRC, and tumor budding are shown in Table 2. High-grade budding was associated with T stage (*p* < 0.001), N stage (*p* < 0.001), TNM stage (*p* < 0.001), MMR status (*p* < 0.05), venous invasion (*p* < 0.001), KM grade (*p* < 0.001), and TSP (*p* < 0.001).

**Table 2:** The relationship between tumor budding and clinicopathological features of patients with primary operable colorectal cancer

	Tumor budding [*N* (%)]		*p* value
	Low [684 (72%)]	High [268 (28%)]	
*Host characteristics*			
Age (years)			0.110
< 65	205 (30)	92 (34)	
> 65	479 (70)	176 (66)	
Sex			0.199
Male	334 (49)	122 (46)	
Female	350 (51)	146 (54)	
*Tumor characteristics*			
Tumor site			0.451
Colon	511 (75)	202 (75)	
Rectum	173 (25)	66 (25)	
T stage			< 0.001
I	38 (6)	2 (1)	
II	101 (15)	16 (6)	
III	393 (57)	121 (45)	
IV	152 (22)	129 (48)	
N stage			< 0.001
0	471 (69)	111 (42)	
1	154 (23)	99 (37)	
2	55 (8)	57 (21)	
TNM stage			< 0.001
1	120 (17)	11 (4)	
2	349 (51)	96 (36)	
3	209 (31)	146 (55)	
4	6 (1)	15 (5)	
Proliferation index			0.285
Low	318 (47)	131 (49)	
High	357 (53)	134 (51)	
Mismatch repair status			0.012
Competent	539 (81)	224 (87)	
Deficient	129 (19)	33 (13)	
BRAF status			0.066
Low	519 (78)	216 (83)	
High	143 (22)	44 (17)	
Necrosis			0.499
< 25%	417 (62)	164 (61)	
> 25%	259 (38)	103 (39)	
Venous invasion			< 0.001
Absent	493 (72)	138 (52)	
Present	191 (28)	130 (48)	
Klintrup–Makinen			< 0.001
Weak	432 (64)	219 (82)	
strong	248 (36)	48 (18)	
Tumor stroma percentage			< 0.001
≤ 50	542 (81)	162 (61)	
> 50	127 (19)	105 (39)	

**Fig. 3:**
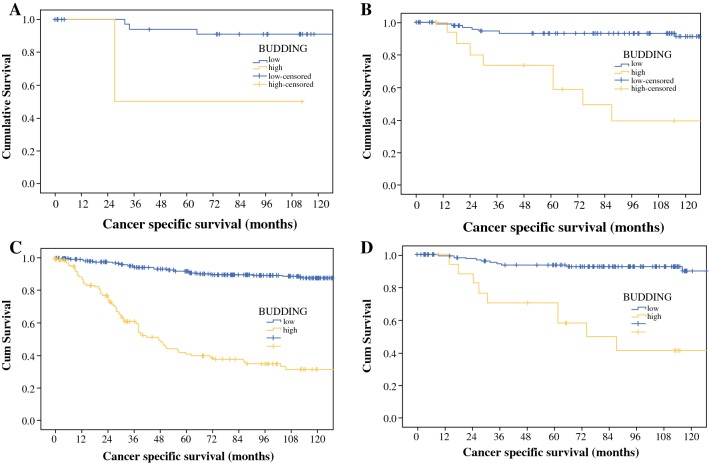
Relationship between tumor budding and cancer-specific survival in **a** T stage 1, **b** T stage 2, **c** T stage 3, and **d** T stage 4 primary operable colorectal cancer (*p* < 0.05). *Cum* cumulative

The relationship between T stage (1–4), clinicopathological characteristics, and CSS in patients with primary operable CRC are shown in Table 3 and Fig. 3a–d. T1 and T2 stages were associated with tumor budding (*p* < 0.01 and *p* < 0.001, respectively), and T3 stage was associated with venous invasion (*p* < 0.01), Ki-69 proliferation index (*p* < 0.01), KM grade (*p* < 0.05), and tumor budding (*p* < 0.001). T4 stage was associated with N stage (*p* < 0.05), venous invasion (*p* < 0.05), MMR status (*p* < 0.05), and tumor budding (*p* < 0.001).

**Table 3:** Relationship between T stage, clinicopathological characteristics, and cancer-specific survival in patients with primary operable colorectal cancer

T stage	Univariate analysis	*p* value	Multivariate analysis	*p* value
*T1 [n = 40]*				
N stage (0/1/2)	1.55 (0.16–14.92)	0.704		
Ki67 proliferation Index (low/high)	1.66 (0.17–15.98)	0.660		
Mismatch repair status (competent/deficient)	1.44 (0.14–13.85)	0.753		
BRAF status (low/high)	0.04 (0–308.53)	0.569		
Tumor necrosis (low/high)	0.31 (0.00–375.83)	0.468		
Venous invasion (no/yes)	0.46 (0.0–17,931)	0.760		
Klintrup–Makinen grade (weak/strong)	0.142 (0.015–1.37)	0.091	0.97 (0.008–1.19)	0.068
Tumor stroma percentage (low/high)	0.043 (0.00–17,062)	0.685		
Tumor budding (low/high)	8.99 (0.92–88.13)	0.059	8.44 (1.12–303.95)	0.042
*T2 [n = 117]*				
N stage (0/1/2)	1.92 (0.88–4.20)	0.104		
Ki67 proliferation index (low/high)	0.557 (0.26–2.02)	0.547		
Mismatch repair status (competent/deficient)	0.75 (0.17–3.33)	0.704		
BRAF status (low/high)	1.21 (0.34–4.33)	0.78		
Tumor necrosis (low/high)	1.41 (0.48–4.13)	0.530		
Venous invasion (no/yes)	3.18 (1.12–9.02)	0.030	2.78 (0.96–8.11)	0.060
Klintrup–Makinen grade (weak/strong)	0.60 (0.22–1.66)	0.328		
Tumor stroma percentage (low/high)	1.41 (0.48–4.13)	0.530		
Tumor budding (low/high)	8.39 (3.03.03–23.20)	< 0.001	7.86 (2.81–21.95)	< 0.001
*T3 [n = 514]*				
N stage (0/1/2)	1.74 (1.36–2.22)	< 0.001	1.20 (0.92–1.56)	0.176
Ki67 proliferation index (low/high)	0.98 (0.97–0.99)	0.002	0.57 (0.38–0.87)	0.008
Mismatch repair status (competent/deficient)	0.87 (0.50–1.50)	0.610		
BRAF status (low/high)	0.96 (0.60–1.56)	0.882		
Tumor necrosis (low/high)	1.04 (0.70–1.54)	0.848		
Venous invasion (no/yes)	1.78 (1.21–2.62)	0.003	1.74 (1.16–2.60)	0.007
Klintrup–Makinen grade (weak/strong)	0.37 (0.21–0.65)	0.001	0.55 (0.30–0.99)	0.049
Tumor stroma percentage (low/high)	1.75 (1.15–2.66)	0.008	0.96 (0.61–1.53)	0.877
Tumor budding (low/high)	8.98 (4.03–9.39)	< 0.001	9.11 (5.92–14.03)	< 0.001
*T4 [n = 281]*				
N stage (0/1/2)	2.07 (1.62–2.65)	< 0.001	1.34 (1.02–1.76)	0.032
Ki67 proliferation index (low/high)	0.78 (0.57–1.18)	0.246		
Mismatch repair status (competent/deficient)	0.21 (0.84–0.51	0.001	0.32 (0.13–0.80)	0.015
BRAF status (low/high)	0.84 (0.49–1.41)	0.504		
Tumor necrosis (low/high)	0.93 (0.63–1.39)	0.736		
Venous invasion (no/yes)	2.99 (1.98–4.52)	< 0.001	1.89 (1.22–2.95)	0.005
Klintrup–Makinen grade (weak/strong)	0.35 (0.19–0.63)	<0.001	0.62 (0.33–1.14)	0.124
Tumor stroma percentage (low/high)	0.99 (0.98–1.01)	0.505		
Tumor budding (low/high)	10.36 (5.96–18.01)	< 0.001	8.23 (4.59–14.73)	< 0.001

The relationship between TNM stage, clinicopathological characteristics, and CSS in patients with primary operable CRC are shown in Table 4. TNM stages I and II were associated with venous invasion (*p* < 0.05), KM grade (*p* < 0.05), and tumor budding (*p* < 0.001). TNM stage III was associated with venous invasion (*p* < 0.05) and tumor budding (*p* < 0.001), and TNM stage IV was associated with tumor budding (*p* < 0.05).

**Table 4:** Relationship between TNM stage, clinicopathological characteristics, and cancer-specific survival in patients with primary operable colorectal cancer

TNM stage	Univariate analysis	*p* value	Multivariate analysis	*p* value
*Stage 1 [n = 131]*				
Ki67 proliferation index (low/high)	0.65 (0.22–1.95)	0.445		
Mismatch repair status (competent/deficient)	0.39 (0.0–21.82)	0.313		
BRAF status (low/high)	0.57 (0.07–4.38)	0.585		
Tumor necrosis (low/high)	1.69 (0.57–5.19)	0.353		
Venous invasion (absent/present)	3.44 (1.05–11.22)	0.041	3.53 (1.02–12.17)	0.046
Klintrup–Makinen grade (weak/strong)	0.28 (0.08–0.89)	0.032	0.27 (0.08–0.94)	0.040
Tumor stroma percentage (low/high)	1.79 (0.49–6.50)	0.378		
Tumor budding (low/high)	9.96 (3.34–29.72)	< 0.001	7.90 (2.63–23.74)	< 0.001
*Stage 2 [n = 445]*				
Ki67 proliferation index (low/high)	0.55 (0.34–0.89)	0.014		
Mismatch repair status (competent/deficient)	0.58 (0.29–1.14)	0.113		
BRAF status (low/high)	0.97 (0.55–1.73)	0.927		
Tumor necrosis (low/high)	0.87 (0.53–1.40)	0.555		
Venous invasion (absent/present)	2.34 (1.47–3.74)	0.001	2.05 (1.27–3.31)	0.003
Klintrup–Makinen grade (weak/strong)	0.41 (0.22–0.77)	0.005	0.51 (0.26–0.97)	0.040
Tumor stroma percentage (low/high)	3.07 (1.89–4.98)	0.001	1.53 (0.92–2.54)	0.102
Tumor budding (low/high)	10.21 (6.26–16.64)	< 0.001	10.23 (6.12–17.07)	< 0.001
*Stage 3 [n = 355]*				
Ki67 proliferation index (low/high)	0.66 (0.46–0.95)	0.025	0.99 (0.99–1.00)	0.296
Mismatch repair status (competent/deficient)	0.83 (0.45–1.50)	0.529		
BRAF status (low/high)	0.83 (0.52–1.34)	0.453		
Tumor necrosis (low/high)	1.29 (0.90–1.85)	0.166		
Venous invasion (absent/present)	1.81 (1.27–2.59)	0.001	1.55 (1.08–2.23)	0.017
Klintrup–Makinen grade (weak/strong)	0.46 (0.28–0.75)	0.0502	0.62 (0.38–1.01)	0.054
Tumor stroma percentage (low/high)	1.27 (0.87–1.85)	0.218		
Tumor budding (low/high)	7.46 (4.87–11.42)	< 0.001	9.11 (5.92–14.03)	< 0.001
*Stage 4 [n = 21]*				
Ki67 proliferation index (low/high)	0.58 (0.21–1.61)	0.294		
Mismatch repair status (competent/deficient)	4.27 (0.48–38.24)	0.195		
BRAF status (low/high)	0.72 (0.23–2.21)	0.561		
Tumor necrosis (low/high)	0.63 (0.18–2.23)	0.478		
venous invasion (absent/present)	1.28 (0.47–3.47)	0.628		
Klintrup–Makinen grade (weak/strong)	0.31 (0.0–13.67)	0.264		
Tumor stroma percentage (low/high)	1.48 (0.55–3.99)	0.444		
Tumor budding (low/high)	10.82 (1.38–85.02)	0.024	10.82 (1.38–85.02)	0.024

Tumor budding is an independent prognostic factor in all stages with venous invasion, independent of stages I–III, and KM grade, independent of stages I–II.

## Discussion

The results of the present study show that tumor budding effectively stratifies CSS in patients with primary operable cancer. Furthermore, compared with other tumor characteristics, including T and nodal stage, tumor budding had the highest hazard ratio, approximately three times that of any other tumor characteristic. Therefore, tumor budding is a good candidate to form the basis of a new staging system for primary operable CRC.

It was also of interest that in T1 and T2 tumors, only tumor budding had significant prognostic value. This would indicate that tumor budding occurs early in tumor invasion and may represent aggressive characteristics of malignant tumors, such as loss of cell adhesion and local invasion.^5^,^20^ Indeed, it may be argued that given its association with other characteristics of the tumor and its microenvironment, tumor budding is the most important component of tumor invasion. Indeed, the prognostic importance of tumor budding in local excision specimens in predicting outcome and/or predictive of nodal metastatic disease in stage pT1 CRCs is increasingly being recognized.^1^,^16^,^17^,^20^

The Ki-67 antigen is widely used to evaluate tumor proliferative activity as autonomous cell proliferation is a main feature of neoplasia. However, despite the clear association of tumor budding with migration and invasion, paradoxically, tumor buds appear not to be associated with proliferation. However, the association between tumor budding intensity and proliferative activity is still poorly understood and it is speculated that host invasion, by budding tumor cells, might be activated only after the cell cycle has been switched off.^2^,^15^ Further work is required to elucidate the molecular basis of this relationship.

In the present study, it was of interest that other components of the tumor microenvironment had increasing prognostic value in patients with T3 and T4 disease. The basis of this observation is not clear, however it may be that in larger, more invasive tumors, the components of the tumor microenvironment, such as the tumor stroma and inflammatory infiltrate, become more important in determining the future of the primary tumor. For example, the good outcome associated with a pronounced tumor inflammatory cell infiltrate may be due to the effective elimination of the tumor. In contrast, the poor outcome associated with the pronounced tumor stroma may be due to the supportive environment for the tumor. Indeed, Ueno et al. reported an association between fibrotic immature stroma and the intensity of tumor budding as histological dedifferentiation, including dissociation of cancer cells and the first step of invasion. Therefore, the present findings further support a pertinent role of tumor stroma in facilitating tumor cell de-differentiation and dissemination. Further work is required to elucidate the molecular basis of this relationship.

## Conclusions

The findings of the present study indicate that tumor budding effectively stratifies cancer survival in patients with primary operable CRC. This stratification is independent of recognized tumor factors, including TNM stage. Therefore, the ITBCC budding evaluation method should be used to assess tumor budding and may form the basis of a new staging system in patients with CRC.
